# Assessing Adolescent Asthma Symptoms and Adherence Using Mobile Phones

**DOI:** 10.2196/jmir.2413

**Published:** 2013-07-17

**Authors:** Shelagh A Mulvaney, Yun-Xian Ho, Cather M Cala, Qingxia Chen, Hui Nian, Barron L Patterson, Kevin B Johnson

**Affiliations:** ^1^School of NursingVanderbilt University School of MedicineNashville, TNUnited States; ^2^Department of Biomedical InformaticsVanderbilt University School of MedicineNashville, TNUnited States; ^3^Department of PediatricsVanderbilt University School of MedicineNashville, TNUnited States; ^4^School of MedicineUniversity of AlabamaBirmingham, ALUnited States; ^5^Department of BiostatisticsVanderbilt University School of MedicineNashville, TNUnited States

**Keywords:** asthma, adherence, mobile technology, adolescent, assessment

## Abstract

**Background:**

Self-report is the most common method of measuring medication adherence but is influenced by recall error and response bias, and it typically does not provide insight into the causes of poor adherence. Ecological momentary assessment (EMA) of health behaviors using mobile phones offers a promising alternative to assessing adherence and collecting related data that can be clinically useful for adherence problem solving.

**Objective:**

To determine the feasibility of using EMA via mobile phones to assess adolescent asthma medication adherence and identify contextual characteristics of adherence decision making.

**Methods:**

We utilized a descriptive and correlational study design to explore a mobile method of symptom and adherence assessment using an interactive voice response system. Adolescents aged 12-18 years with a diagnosis of asthma and prescribed inhalers were recruited from an academic medical center. A survey including barriers to mobile phone use, the Illness Management Survey, and the Pediatric Asthma Quality of Life Questionnaire were administered at baseline. Quantitative and qualitative assessment of asthma symptoms and adherence were conducted with daily calls to mobile phones for 1 month. The Asthma Control Test (ACT) was administered at 2 study time points: baseline and 1 month after baseline.

**Results:**

The sample consisted of 53 adolescents who were primarily African American (34/53, 64%) and female (31/53, 58%) with incomes US$40K/year or lower (29/53, 55%). The majority of adolescents (37/53, 70%) reported that they carried their phones with them everywhere, but only 47% (25/53) were able to use their mobile phone at school. Adolescents responded to an average of 20.1 (SD 8.1) of the 30 daily calls received (67%). Response frequency declined during the last week of the month (*b*=-0.29, *P*<.001) and was related to EMA-reported levels of rescue inhaler adherence (*r*= 0.33, *P*=.035). Using EMA, adolescents reported an average of 0.63 (SD 1.2) asthma symptoms per day and used a rescue inhaler an average of 70% of the time (SD 35%) when they experienced symptoms. About half (26/49, 53%) of the instances of nonadherence took place in the presence of friends. The EMA-measured adherence to rescue inhaler use correlated appropriately with asthma control as measured by the ACT (*r*=-0.33, *P*=.034).

**Conclusions:**

Mobile phones provided a feasible method to assess asthma symptoms and adherence in adolescents. The EMA method was consistent with the ACT, a widely established measure of asthma control, and results provided valuable insights regarding the context of adherence decision making that could be used clinically for problem solving or as feedback to adolescents in a mobile or Web-based support system.

## Introduction

Treatment for asthma is typically addressed initially through the use of what is known as a “rescue” inhaler that is used at the time that symptoms, such as shortness of breath, cough, or wheezing, occur. If symptoms persist over time despite use of rescue medications, a controller or “everyday” inhaler is prescribed. Thus, adherence to use of inhalers is a critical mediator of asthma control and health care utilization [1]. However, the primary method for measuring medication adherence, patient self-report, is hindered by recall error, response bias, and difficulty identifying specific contextual and psychosocial barriers to adherence [2]. In pediatric asthma, Jonasson and colleagues (1999) found that as adherence decreased (according to remaining doses in inhalers) traditional retrospective self-report became increasingly inaccurate [3]. Other research has documented overreporting of asthma control by adolescents using traditional retrospective self-report measures compared to clinician-rated estimates of asthma control [4].

A growing body of literature has documented progress in measurement of adherence and health behaviors through real-time or near real-time data capture using mobile technologies. This measurement method is known as ecological momentary assessment or EMA [2]. The method may reduce response bias introduced through social desirability and/or retrospective self-report and allows in vivo assessment of emotions, behaviors, and cognitions actually in, or near, the context in which they are experienced. This approach has been used to assess a wide variety of health behaviors and health-related conditions [5,6], pain [7,8], adolescent diabetes [9], and smoking [10], as well as to assess health information needs [11]. This method allows assessment of many aspects of health and health behaviors including physiological processes, timing or duration of events, patterns of symptoms over time, and situational and psychosocial conditions surrounding a health decision or event [2,12].

Psychosocial barriers to adherence for the adolescent age group often involve peers and feelings of stigma related to using an inhaler or taking medications [13]. Identification of the behavioral and situational correlates of adherence is necessary for clinicians and parents to engage the adolescent in focused problem solving. Mobile assessment has been qualitatively explored as a potential method to monitor asthma [14], but no research has explored this technology to assess adherence, symptoms, or used it to identify situational correlates of asthma adherence such as social context.

The primary goals of this research were to determine the feasibility of using EMA to assess asthma symptoms and adherence, identify contextual correlates of adherence using EMA, and compare EMA to the Asthma Control Test (ACT), a widely used self-report assessment of asthma control.

## Methods

### Participants

Participants were recruited from Vanderbilt University Medical Center. Adolescent candidates were included if they were between the ages of 12-18 years, had use of their own cell phone, were prescribed an inhaler, and had a diagnosis of asthma, as indicated by their parent or guardian (referred to as “parent”).

Potential participants with a diagnosis of asthma were identified in the General Pediatrics clinic database by the director (co-author, BP) through use of ICD-9 (International Classification of Diseases, ninth revision) codes and reviews of patient medication lists. All participants were receiving primary care through the Vanderbilt Medical Center. We used three recruitment strategies. First, the research team sent letters to parents of adolescents (N=515) determined to be eligible for participation based on a diagnosis of asthma present in the adolescents’ medical records. If an adolescent was 18 years or older, the letter was addressed directly to him/her. Following the letters, parents of the adolescent candidate were contacted to determine interest. Second, we distributed flyers and interest cards to the waiting areas of General Pediatrics, Adolescent Medicine, and Pediatric Pulmonology clinics, as well as the pediatric emergency department. A research assistant contacted candidates who completed and submitted an interest card. Finally, we posted an announcement to a research notification listserv and in a medical center online newsletter. A total of 199 candidate parent-adolescent dyads were assessed for eligibility via telephone call with the parent. We identified 83 candidates out of 199 (42%) who were not eligible to participate or not interested in participating and 116 candidates (58%) who were eligible to participate. Candidates were primarily excluded because the adolescent did not have their own cell phone (22/83, 27%) or lacked interest in the study (28/83, 34%). Out of those who were eligible, 54 (47%) were enrolled and 62 (53%) did not progress to baseline because a parent could not be reached, or the family did not show up for their initial appointment. Out of the candidates who were enrolled, 1 participant did not complete the study. The final dataset consisted of 53 adolescent-guardian dyads that completed the study.

### Procedures and Measures

At the initial session, adolescents were informed that parents and clinicians would not have access to their study data, nor would any indication of their participation be included in their medical record. At baseline, adolescents completed questions generated for this research related to mobile phone use and possible barriers to adolescent mobile phone use for asthma (ie, where they were able to use their phones, typical response to calls, need to earn their phone use), the ACT, the Illness Management Survey (IMS), and the Pediatric Asthma Quality of Life Questionnaire (PAQLQ). The ACT is a widely used 5-item measure of symptoms and rescue inhaler use over the previous 4 weeks [15]. The measure has been validated for use over the telephone [16]. We also included an item to assess the use of a daily controller inhaler over the previous 4 weeks. Parents completed the ACT as it related to their adolescent at baseline. Higher scores indicate better asthma control.

The IMS consists of 27 items that measure perceptions of barriers to adherence in adolescents with chronic illness including interactions with health care providers, cognitive abilities, family/peer influences, and denial of the illness. Responses range from strongly disagree (1) to strongly agree (5). The measure has been validated in adolescents with asthma and has demonstrated adequate internal reliability (Cronbach alpha=.84) [17]. Higher scores indicate greater perceived barriers to adherence.

The PAQLQ is a widely used measure of the perception of the impact of asthma on daily life. The PAQLQ is related to an objective measure of asthma control (spirometry) and has adequate psychometric properties cross-sectionally and at test-retest [18]. We used the brief version of the instrument with 13 items. Higher scores indicate greater perceived quality of life. All survey data were collected and managed using the online survey system Research Electronic Data Capture (REDCap) [19]. Parent consent and adolescent assent were obtained before study procedures commenced. The Institutional Review Board approved all study procedures.

**Figure 1:**
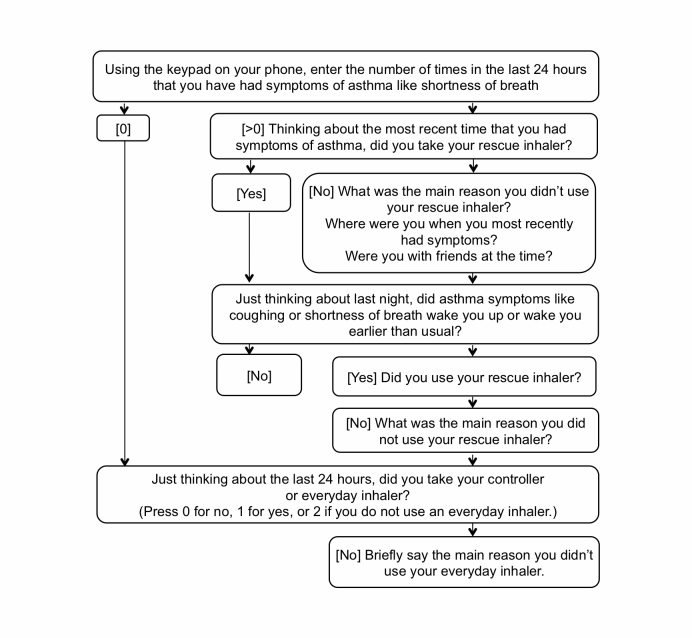
Ecological momentary assessment administered through mobile phones.

Following baseline procedures, EMA was used to measure symptoms and adherence. An interactive voice response system (Telesage) was administered through the adolescents’ mobile phones. At baseline, each adolescent completed a practice call on their cell phone with the researcher present to ensure they understood how to respond to questions. Each participant received 1 call per day for 30 days. The calls were scheduled for the family’s preferred time between 6-8 p.m. in the evening. Adolescents could return a call to the system (“incoming”) before midnight that day if they missed the “outgoing” call to them. The automated adaptive phone survey included between 2-9 questions depending on responses to the questions and lasted between 15-90 seconds. Figure 1 shows the adaptive EMA items. Adolescents responded to numeric or yes/no items using the keypad on their mobile phone and qualitative questions by voice.

After 1 month of EMA, adolescents and their guardians were asked to complete the ACT using REDCap at this second time point (“Time 2”).

### Statistical Analyses

To calculate rescue inhaler adherence for each participant using EMA, we divided the number of days the inhaler was used by the number of days asthma symptoms were reported. Adolescents who reported having no asthma symptoms did not receive a rescue adherence score. To calculate controller (everyday) adherence for the EMA method, we divided the number of days participants reported using their controller inhaler by the total number of daily call responses. A paired *t* test was used to determine if there were any differences between adolescents’ ACT scores reported at baseline and Time 2 and their parents’ reported scores. Data from the ACT administered at Time 2 were used for these comparisons in order to correspond to the calling period. Only EMA-measured rescue inhaler use was compared to the ACT, as it measures control of asthma related to rescue inhaler use. Within-subject comparisons of adherence and Spearman correlations were conducted to examine the relationship between EMA adherence and other self-report measures. Statistics were carried out using Stata v10 and R v2.13.1.

## Results

Characteristics of the sample are in Table 1. The sample was primarily female (31/53, 58%), African American (33/53, 64%), and mid- to low-income with an annual household income at or below US$40,000 (29/53, 55%). The mean IMS score for assessing barriers to adherence was 2.7 out of 5 (SD* *0.6) with a lower score indicating lower barriers. Mean PAQLQ score was 5.5 (SD* *1.4) out of a maximum of 7. Mean PAQLQ Symptoms Scale score was 5.4 (SD* *1.3). The mean total ACT score at baseline was 19.8 (SD 4.0, range 9-25), and at Time 2 was 19.4 (SD 4.1, range 11-25). There were no statistically significant differences between adolescent baseline and Time 2 scores (*P*=.36) or between adolescent and parent ACT scores at either baseline or Time 2 (*P*=.36 and .70, respectively).

### Cell Phone Use

Adolescents were very confident about how to use their cell phones (mean 9.1, SD 1.8, range 1-10). Just over half (31/53, 58%) were able to access the Internet with their phones. The majority (50/53, 94%) reported that their school had restrictions on using mobile phones at school with 38% (18/47) not allowed to use the phone at all, and 47% (22/47) of those allowed to use the phone being able to do so only outside of class. The majority of adolescents (28/53, 53%) were not sure if their school would allow mobile phone use for asthma, 23% (12/53) were not allowed, and 25% (13/53) could use their phones specifically for asthma. Most felt that school mobile phone policies did not impact how they took care of asthma (46/53, 87%). Outside of school, 70% of adolescents (37/53) reported that they always take their cell phones with them everywhere they go, and 57% (30/53) of adolescents reported that they immediately try to see who contacted them upon receiving a call or message. About one-third of adolescents were required to earn or pay for their cell phone plans (16/53, 30%). Adolescents reported relatively little use of their phones for asthma, such as accessing online information (9/53, 17%), sending a text message (9/53, 17%), setting a reminder to do something about asthma (17/53, 32%), calling a family member (21/53, 40%), or talking to a nurse or doctor (10/53, 19%). None reported using a smartphone application for asthma.

### Disposition of Daily Calls and Missing Data

**Figure 2:**
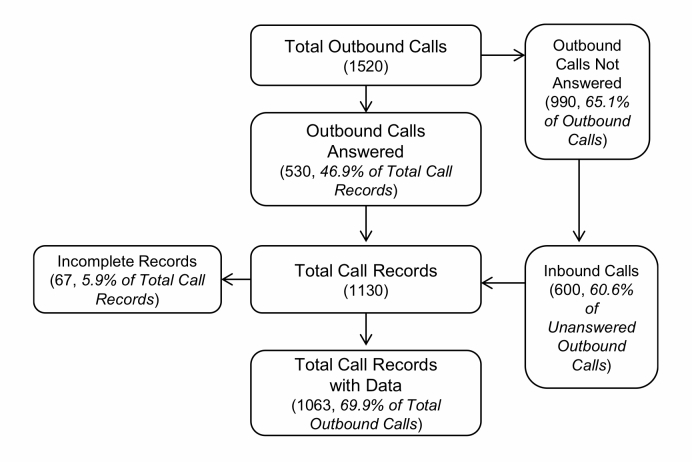
Flow diagram of daily EMA calls.

Figure 2 summarizes the disposition of daily calls. Figure 2 shows that there were 69.9% (1063/1520) of calls with data available for analyses. This included outgoing and incoming calls (returned outgoing calls). About 6% (67/1130) of the calls were excluded due to dropped calls or hang-ups.

**Figure 3:**
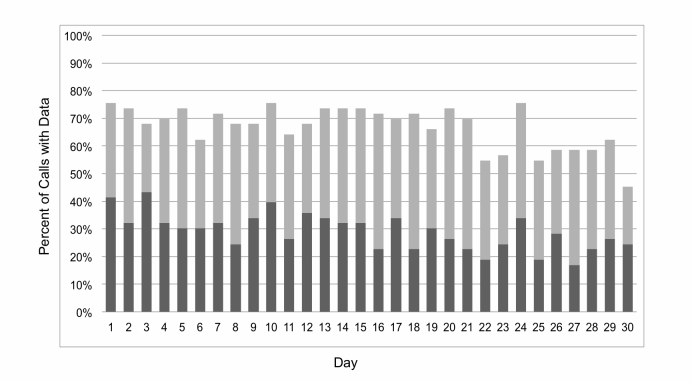
Percent of calls with data per day over 30 days (Dark gray=outbound calls; light gray=inbound calls).

Figure 3 shows that an average of 70% of calls each day over the study period contained complete data. Adolescents had an average of 20.1 (SD 8.1) responses or 67% (SD 27.7) of all daily call responses each. There was some decline in calls with data over time (*b*=-0.29, *P*<.001). Missingness was defined as the number of days of no response to the calls. Missingness was not correlated with age, gender, household income, asthma control as measured by the ACT or controller adherence as measured by EMA (*P*=.86, .41, .10, .94, .12, respectively). However, missingness was significantly related to EMA-measured rescue inhaler adherence (*r*=-.33, *P*=.04), indicating that as the number of missed calls increased, adherence decreased.

**Table 1:** Characteristics of the sample (n=53).

Characteristics		Mean (SD) or n (%)
Age		15.2 (1.7)
**Gender**		
	Male	22 (42%)
	Female	31 (58%)
**Ethnicity**		
	White/Caucasian	18 (34%)
	African American	33 (62%)
	Hispanic	2 (4%)
**Type of school**		
	Public	49 (92%)
	Private	2 (4%)
	Home-schooled	2 (4%)
**Home life**		
	Single parent	26 (49%)
	Both parents	23 (43%)
	Other legal guardian	4 (8%)
**Household income (USD)**		
	Less than $20,000	17 (32%)
	$20,001-$40,000	12 (23%)
	$40,001-$70,000	12 (23%)
	More than $70,000	8 (15%)
	Decline to answer	4 (8%)
**Guardian’s education level**		
	Grade 7-11	8 (15%)
	Grade 12	11 (21%)
	Some college, no degree	16 (30%)
	College degree	13 (25%)
	Graduate school	5 (9%)
**Asthma medication regimen**		
	Rescue inhaler	53 (100%)
	Controller & rescue inhaler	23 (43%)

### Symptoms, Adherence, and Contextual Characteristics

Of the 1063 calls with data, 27% (287/1063) of those calls recorded symptoms of asthma during the previous 24 hours. During the study period, 12 of the 53 (23%) adolescents reported having no asthma symptoms. When adolescents reported a symptom, they reported on average using a rescue inhaler 70% (SD 35) of the time. The most common reasons reported by adolescents for not using the rescue inhaler included not needing the inhaler (23/57, 40%) or not having it (17/57, 30%). When symptoms were experienced, adolescents were most commonly at home (18/51, 32%), at school (8/51, 16%), or exercising (6/51, 12%). Adolescents reported being in the company of friends 53% (29/55) of the time that the rescue inhaler was not used in the context of symptoms. Controller inhaler use averaged 80% of the time over the study period (SD 29). The most common reason adolescents reported for not using the controller was they felt they did not need it (135/207, 65%).

### Relationship Between EMA and ACT

Table 2 shows summary statistics for and bivariate correlations between the ACT and EMA. The ACT total and EMA means were appropriately negatively correlated indicating that as symptoms and use of a rescue inhaler increased, asthma control decreased. Individual ACT and EMA items also exhibited significant positive correlations on the three corresponding items related to shortness of breath, nighttime symptoms, and rescue inhaler use.

**Table 2:** Comparisons of EMA-measured rescue inhaler adherence and ACT scores for totals and corresponding items.

EMA	Median, mean (SD)	ACT	Median, mean (SD)	*r* ^a^
Rescue inhaler adherence	0.83, 0.70 (0.35)	Total score for all 5 items^b^	19, 18.5 (4.0)	-0.33^c^
Using the keypad on your phone, enter the number of times, in the last 24 hours, that you have had symptoms of asthma, like shortness of breath or coughing.	0.25, 0.63 (1.2)	During the past 4 weeks, how often have you had shortness of breath?	2, 2.5 (1.3)	0.44^d^
Just thinking about last night, did asthma symptoms like coughing or shortness of breath wake you up, or wake you earlier than usual? ^c^	0, 0.06 (0.10)	During the past 4 weeks, how often did your asthma symptoms (wheezing, coughing, shortness of breath, tightness or pain) wake you up at night or earlier than usual in the morning?	1, 1.8 (1.2)	0.65^d^
Thinking about the most recent time that you had symptoms of asthma in the last 24 hours, did you take your rescue inhaler?^e^	0.12, 0.20 (0.20)	During the past 4 weeks, how often have you used your rescue inhaler or nebulizer medication (such as albuterol)?	2, 2.4 (1.2)	0.52^d^

^a^EMA responses scaled to ACT for comparison.

^b^Total ACT score ranges from 5 to 25.

^c^ *P*=.034.

^d^ *P*<.001.

^e^“Yes” scored as 1, “No” scored as 0.

## Discussion

### Principal Results

We investigated the feasibility of a mobile method of measuring asthma symptoms and adherence in adolescents over time and compared that to a widely used self-report measure of asthma control. Regarding access and use of mobile phones, we determined that neither mobile phone accessibility nor need for immediate response were barriers to utilizing the phone for health assessment. Just over one third of adolescents were not allowed to use their phones at school at all, but the majority did not view school policies as a barrier to taking care of asthma. Interestingly, only a minority of the sample reported currently using their mobile phone to help with asthma (setting reminders, text messages, use of websites, or mobile apps), and 42% did not have Internet access through their phone. Because school access was unclear, we scheduled daily calls for the evening hours. It appears that real-time assessment and support for asthma using mobile phones could be hindered by current public school policies. In order to address this barrier, it may be necessary for researchers, parents, and advocacy groups to collaborate with school administrators to facilitate the use of mobile health devices for students.

Missing data and response burden are potential issues in daily or momentary health behavior assessments. On average, there was adequate response to the daily calls. Allowing incoming calls greatly enhanced the daily response levels. There was a decline in response to the calls over one month, with the 4th week the point at which that decline was observable. Although a 30-day period was selected to correspond to the established asthma control self-report measure, a 10-20 day assessment period may be optimal for once-a-day assessments in this population [20]. Similar to a previous study in asthma that assessed peak flow values [5], we found a wide range of response levels. It is currently unclear what number of observations should be used to provide the best sampling of asthma symptoms and behaviors. We anticipate that a threshold for adequate sampling will vary by goal of the research and exposure to asthma triggers. Interestingly, momentary assessment using self-report was still susceptible to response bias in terms of missing data. Higher levels of missing data were related to lower adherence. This speaks to the need for unobtrusive or passive methods of adherence assessment.

Measurement of symptoms and adherence using the EMA method provided specific data that may be useful for patient and clinical pattern recognition, problem solving, and decision making at both the population and individual patient levels. For example, the average number of symptoms per day was 0.6 but individual numbers ranged greatly. This method could be used as part of a baseline or periodic monitoring of symptoms during seasonal exposure to asthma triggers or after modification of a regimen. Additionally, insights regarding the reasons for inadequate adherence are difficult to assess clinically. The location-based data obtained for symptom occurrence were not particularly revealing indicating home, school, and sports events as relevant. However, the presence of peers was a prevalent and potentially impactful barrier to adherence in about half of the instances when a rescue inhaler was not used. Of particular interest was that the most common reason adolescents reported not using their rescue or controller inhalers was because they did not believe they needed it, despite the presence of asthma symptoms. These qualitative findings strongly suggest a valuable focus for patient education and adherence problem solving.

### Limitations

Generalizability of our results is somewhat limited by the small sample of this pilot study. The daily calls could have positively influenced adherence through enhanced self-monitoring and greater awareness of symptoms and adherence. However, previous research in adolescents with diabetes did not indicate an EMA monitoring effect [9]. Similarly, a monitoring effect does not appear to have occurred in this study as the baseline and 1-month ACT scores were equivalent. Additional research will be needed to directly compare mobile methods of assessing asthma symptoms and adherence to other assessment methods, such as parent/spouse report, days of school missed, and other objective methods not directly related to the process of mobile data capture. Finally, we included a modified version of the ACT to allow parallel items for parent report of adolescent asthma control. That measure has not been validated.

### Conclusions

The mobile measurement method described here provided a feasible means to probe patterns of symptoms and adherence over time and provided additional qualitative insights regarding contextual reasons for adherence problems. The use of this and related methods may be more expensive to implement compared to the traditional retrospective self-report [21] and is still susceptible to bias in missing data, but should be explored for its added value in clinical practice and integrated with tailored mobile intervention techniques to improve adherence.

## Abbreviations

ACT: Asthma Control Test
AHRQ: Agency for Healthcare Research and Quality
EMA: ecological momentary assessment
ICD-9: International Classification of Diseases, 9th revision
IMS: Illness Management Survey
PAQLQ: Pediatric Asthma Quality of Life Questionnaire
REDCap: Research Electronic Data Capture
